# An epithelioid vascular tumour involving a thigh vein mimicking a saphenous nerve lesion: a case report

**DOI:** 10.1093/jscr/rjag953

**Published:** 2026-10-22

**Authors:** Gopi Gopalapillai, Benedict Kakala, Jason Miller

**Affiliations:** General Surgery, Royal Brisbane and Women's Hospital, Metro North Health, Herston, QLD 4029, Australia; General Surgery, Royal Brisbane and Women's Hospital, Metro North Health, Herston, QLD 4029, Australia; General Surgery, Royal Brisbane and Women's Hospital, Metro North Health, Herston, QLD 4029, Australia

**Keywords:** epithelioid vascular tumour, epithelioid haemangioma, epithelioid haemangioendothelioma, saphenous nerve, peripheral nerve mimic, case report

## Abstract

A 34-year-old man presented with progressive, debilitating pain radiating from the distal medial thigh to the medial foot. Magnetic resonance imaging demonstrated a 1 cm enhancing lesion along the expected course of the saphenous nerve. At exploration, the lesion was separable from the nerve but intimately associated with adjacent vessels. Histology demonstrated an intravascular epithelioid endothelial neoplasm, initially interpreted as epithelioid haemangioendothelioma. Supplementary immunohistochemistry favoured epithelioid haemangioma, although classical eosinophil-rich morphology was absent and definitive classification remained uncertain. No molecular testing was performed. Staging showed no distant disease. Multidisciplinary review recommended completion wide excision because the initial procedure was non-oncological and margins were not assessable. Re-excision demonstrated no residual tumour. At latest follow-up, the preoperative pain had settled, with substantially improved residual paraesthesia. This case highlights vascular tumours as rare peripheral nerve mimics and the importance of integrating operative findings, specialist pathology and multidisciplinary management.

## Introduction

Epithelioid vascular tumours encompass biologically distinct entities ranging from benign epithelioid haemangioma to epithelioid haemangioendothelioma (EHE), an ultra-rare vascular neoplasm with variable clinical behaviour [1, 2]. Their morphological and immunophenotypic overlap can make classification difficult. Rare reports also show that EHE within a neurovascular compartment may mimic a peripheral nerve lesion or produce focal neuropathy [3–5]. We report an intravascular epithelioid vascular tumour of the distal medial thigh that clinically and radiologically appeared to involve the saphenous nerve, highlighting diagnostic uncertainty and multidisciplinary management after an initial non-oncological excision.

## Case report

A 34-year-old man presented with a five-year history of intermittent distal medial right thigh pain that had progressed markedly over the preceding six months. Pain radiated through the medial calf to the medial foot in the sensory distribution of the saphenous nerve. Lower-limb examination was otherwise normal. Magnetic resonance imaging (MRI) demonstrated an ~1 cm contrast-enhancing lesion along the expected course of the saphenous nerve, 8–10 cm proximal to the knee (Fig. 1).

**Figure 1:**
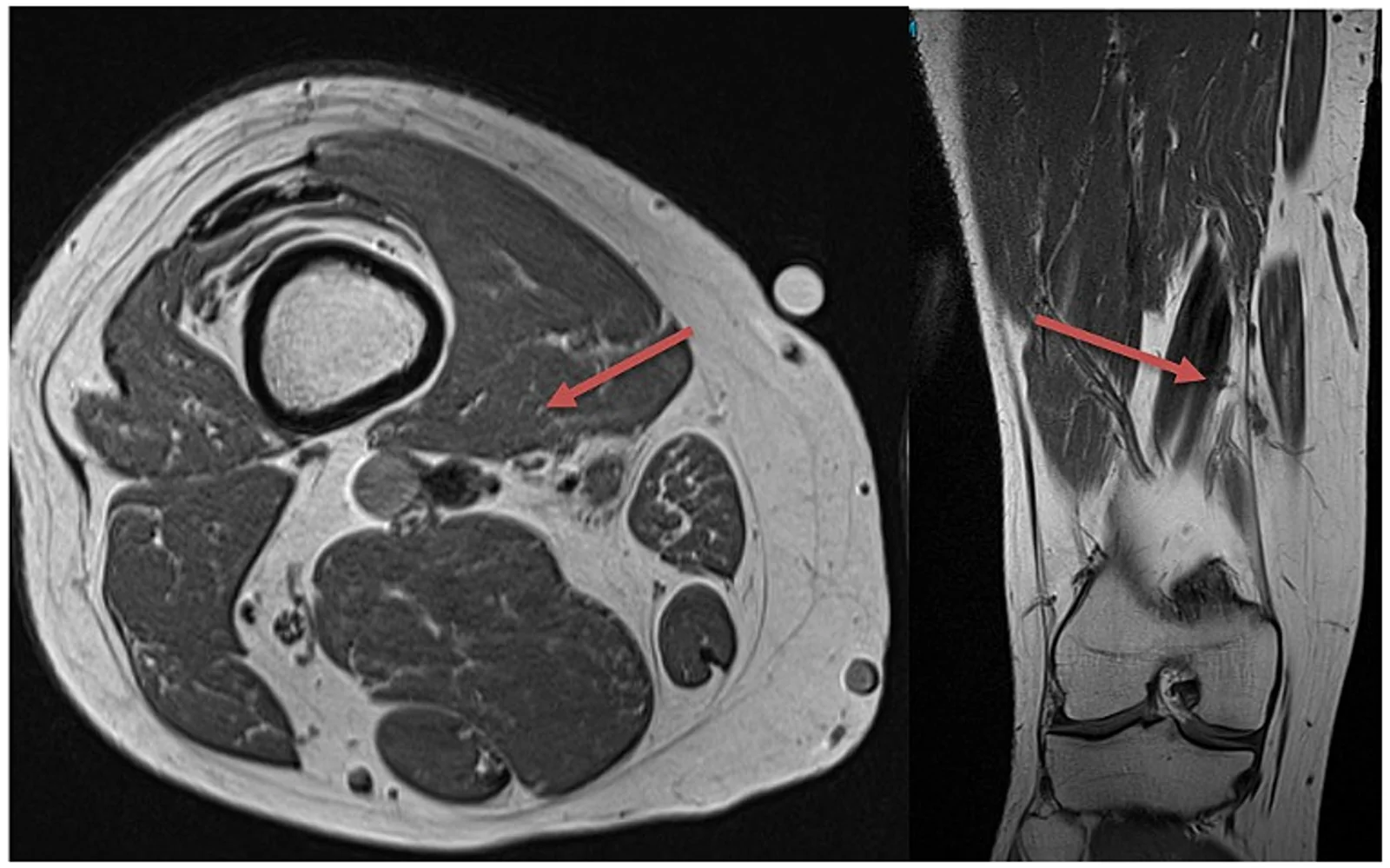
Preoperative MRI of the right distal medial thigh. Axial and coronal T1-weighted images demonstrate the enhancing lesion along the expected saphenous neurovascular plane approximately 8–10 cm proximal to the knee (arrows).

After discussion of observation and operative management, including possible nerve transection and repair if the lesion could not be separated from the nerve, the patient elected surgery. At exploration on 12 November 2025, the saphenous nerve was identified proximally and distally. The lesion was readily separated from the nerve but was intimately related to adjacent vessels, which were controlled and divided. The lesion was excised and the saphenous nerve preserved.

Histology demonstrated an epithelioid endothelial proliferation forming small capillary channels, filling the lumen of a vein, infiltrating the venous wall and extending into surrounding soft tissue. A rare mitosis was present without necrosis. Lesional cells expressed CD31, ERG, CD34, FLI1, and smooth muscle actin, with focal AE1/AE3 expression and a Ki-67 index of approximately 7%–8%. The initial diagnosis was EHE involving a vein. Supplementary immunohistochemistry demonstrated FOSB positivity with retained YAP1, negative CAMTA1 and HHV8, weak diffuse TFE3 staining, and retained INI-1. Specialist pathology review considered the immunophenotype to favour epithelioid haemangioma, although the classical eosinophil-rich inflammatory infiltrate was absent. No molecular testing was performed, and the lesion remained an epithelioid vascular neoplasm of uncertain classification.

Postoperative fluorodeoxyglucose positron-emission tomography-computed tomography (FDG PET CT) showed moderate uptake at the operative site, considered difficult to distinguish from postoperative change, with reactive bilateral inguinal nodes and no distant FDG-avid disease. Inguinal ultrasonography was normal. Repeat MRI on 23 January 2026 showed postoperative change without radiological evidence of residual tumour (Fig. 2).

**Figure 2:**
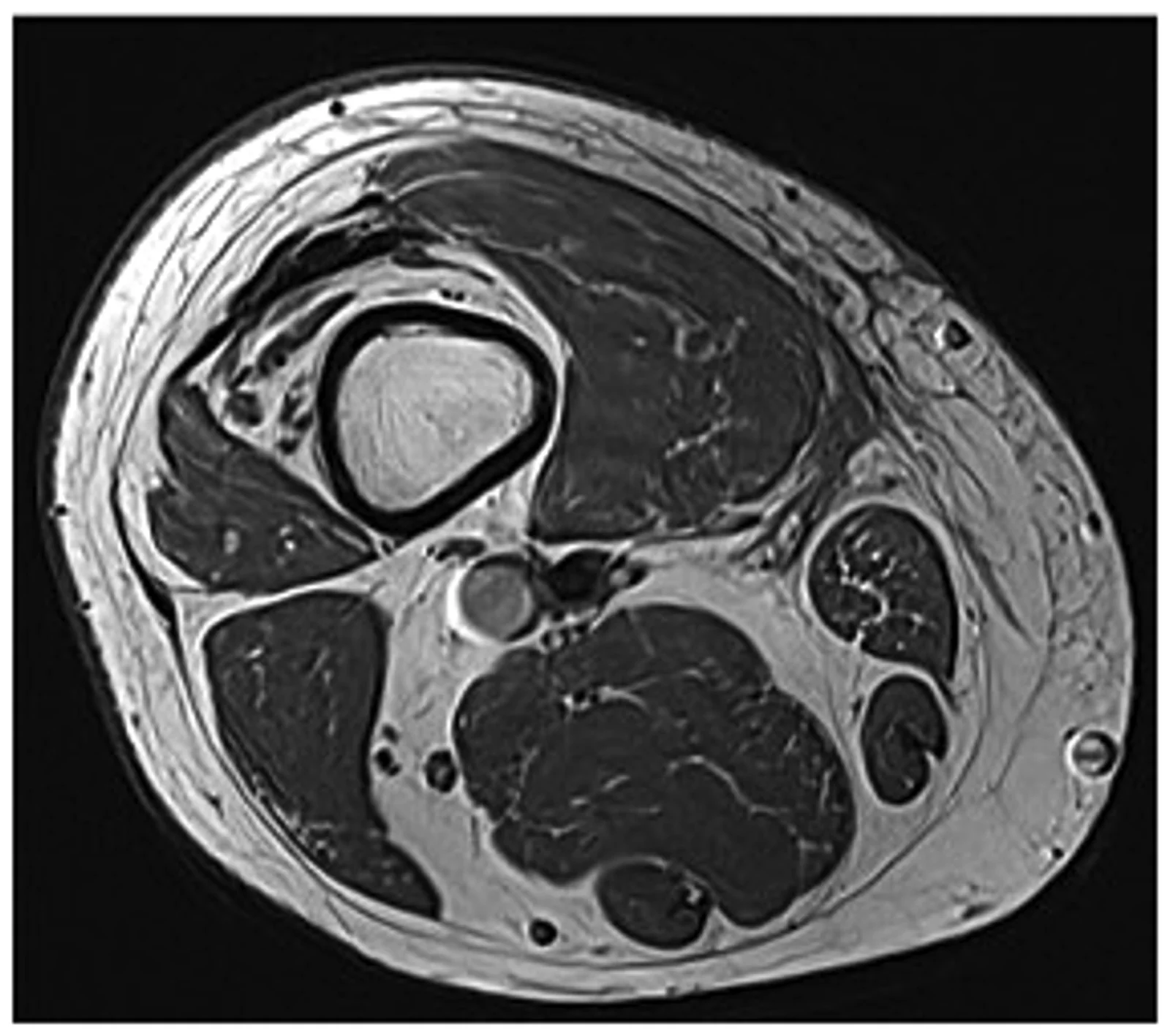
Postoperative MRI of the right thigh demonstrating operative-bed change without a discrete residual lesion.

Multidisciplinary review recommended completion wide excision because of the uncertain biological classification, venous involvement and inability to confirm oncological margins after an operation performed for a presumed nerve lesion. On 20 March 2026 the previous scar and operative cavity were re-excised through deep fascia alongside vastus medialis to the popliteal neurovascular bundle. Extensive initial and supplementary sampling demonstrated postoperative and reparative change only, with no residual lesion identified.

At latest follow-up, the severe preoperative saphenous-distribution pain had settled. Local paraesthesia persisted but was substantially improved. Surveillance was planned with CT chest/abdomen/pelvis at six months and dedicated right-thigh MRI at 12 months.

## Discussion

This case illustrates two linked diagnostic problems: a vascular neoplasm may mimic a peripheral nerve lesion, and epithelioid vascular tumours may remain difficult to subclassify after excision. Similar reports describe EHE involving the upper-arm neurovascular bundle or ulnar artery and presenting as an apparent schwannoma or focal neuropathy [3–5]. In this patient, the symptom distribution and MRI appearance made a peripheral nerve lesion a reasonable preoperative impression, whereas operative separation from the saphenous nerve and subsequent pathology established vascular rather than neural differentiation.

The pathological pathway is summarized in Fig. 3. Intravascular growth and expression of multiple endothelial markers strongly supported a vascular neoplasm. Specialist review confirmed that there were no histological features suggesting a nerve-sheath tumour and no large nerve was present in the biopsy. HHV8 negativity argued strongly against Kaposi sarcoma, while retained INI-1 made epithelioid sarcoma less likely [6–8].

**Figure 3:**
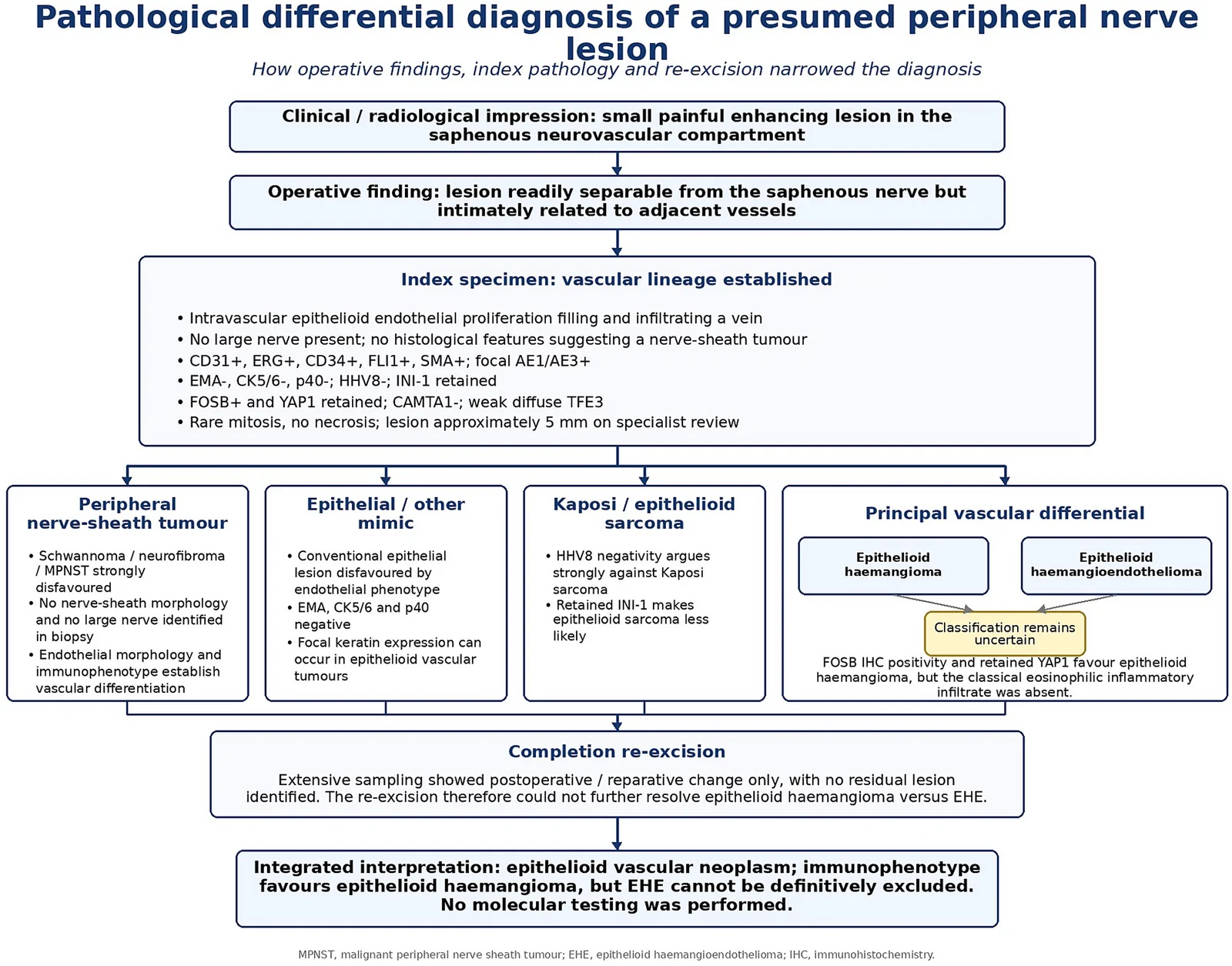
Diagnostic flowchart showing how a presumed peripheral nerve lesion was reclassified as an intravascular epithelioid vascular neoplasm. Supplementary immunohistochemistry favoured epithelioid haemangioma, but classical morphology was absent and EHE could not be definitively excluded. The completion excision showed no residual lesion.

The principal unresolved distinction was epithelioid haemangioma versus EHE. FOSB immunoreactivity and retained YAP1 were considered to favour epithelioid haemangioma, but the absence of the typical eosinophil-rich inflammatory infiltrate prevented classical morphological classification. Most EHEs harbour WWTR1:CAMTA1 fusion, with a smaller YAP1::TFE3 subset, whereas a proportion of epithelioid haemangiomas harbour FOS or FOSB rearrangements [9–12]. Molecular testing was not performed; accordingly, the manuscript retains the diagnostic uncertainty rather than assigning a definitive molecular subtype. Had the lesion represented EHE, specialist review considered its ~5 mm size and low mitotic activity to place it toward the low-grade end of the spectrum. Had the lesion represented EHE, specialist review considered its ~5 mm size and low mitotic activity to place it toward the low-grade end of the spectrum.

Published intravascular lower-limb EHE includes femoral-vein lesions presenting with pain, swelling or presumed thrombosis [13]. The present lesion was smaller and presented predominantly with neuropathic pain. Completion excision was selected despite negative postoperative imaging because the initial procedure was non-oncological and margins were not assessable; for localized technically resectable EHE, consensus recommendations support complete surgical resection; given EHE remained within the differential and the initial margins were not assessable, completion excision was considered appropriate [2]. Re-excision showed no residual tumour and therefore provided no further tissue with which to resolve the original classification. The principal limitation is the absence of definitive molecular classification, but the case remains instructive as an unusual peripheral nerve mimic requiring integration of operative findings, pathology and multidisciplinary oncological management.

## Data Availability

All relevant de-identified clinical information is contained within the article. Additional patient-level data are not publicly available to protect patient privacy.
